# Genetic Pathways Leading to Therapy-Related Myeloid Neoplasms

**DOI:** 10.4084/MJHID.2011.019

**Published:** 2011-05-16

**Authors:** Angela Stoddart, Megan E. McNerney, Elizabeth Bartom, Rachel Bergerson, David J. Young, Zhijian Qian, Jianghong Wang, Anthony A. Fernald, Elizabeth M. Davis, Richard A. Larson, Kevin P. White, Michelle M. Le Beau

**Affiliations:** University of Chicago, Chicago, IL, USA

## Abstract

Therapy-related myeloid neoplasm (t-MN) is a distinctive clinical syndrome occurring after exposure to chemotherapy or radiotherapy. t-MN arises in most cases from a multipotential hematopoietic stem cell or, less commonly, in a lineage committed progenitor cell. The prognosis for patients with t-MN is poor, as current forms of therapy are largely ineffective. Cytogenetic analysis, molecular analysis and gene expression profiling analysis of t-MN has revealed that there are distinct subtypes of the disease; however, our understanding of the genetic basis of t-MN is incomplete. Elucidating the genetic pathways and molecular networks that are perturbed in t-MNs, may facilitate the identification of therapeutic targets that can be exploited for the development of urgently-needed targeted therapies.

## Introduction:

t-MN is a late complication of cytotoxic therapy (radiation and/or chemotherapy) used in the treatment of both malignant and non-malignant diseases.^1^–^4^ Several distinct cytogenetic and clinical subtypes of t-MN are recognized that are closely associated with the nature of the preceding treatment. Patients who develop t-MN following alkylating agent therapy (∼75% of cases) typically show a latency of 3–7 years from alkylating agent exposure (median 5 years), insidious disease onset with an antecedent MDS with peripheral cytopenias, loss, deletion, or rearrangement of chromosomes 5 and/or 7, and a poor prognosis (median survival <8 months).^1^ Typically, all three hematopoietic cell lineages (erythroid, myeloid, and megakaryocytic) are involved in the dysplastic process (trilineage dysplasia), suggesting that the disease arises in a multipotent hematopoietic stem or progenitor cell (HSPC). In contrast, patients who develop t-MN following treatment with drugs targeting topoisomerase II are younger, have a shorter latency period (2–3 years), and present with AML. Balanced translocations involving *MLL* at 11q23, *RUNX1* at 21q22, *CBFB* at 16q22, or *PML* (15q24) and *RARA* (17q12) are common in this subgroup, suggesting that these cytogenetic subsets of t-MN arise in a lineage committed progenitor cell.^3^–^5^ Survival times of t-MN patients are often short, because this disorder is less responsive to current forms of therapy than is AML *de novo*.^1^,^3^,^4^

t-MN represents an important model for cancer. The incidence of t-MN is rising, as a result of the increasing number of cancer survivors at risk of developing this disorder and the changes in therapeutic trends. Secondly, t-MN provides a unique opportunity to examine the effects of mutagens on carcinogenesis in humans, as well as the role of genetic susceptibility to cancer.^3^ Finally, the mechanisms of leukemogenesis that are uncovered in t-MN will likely apply to those subtypes of AML *de novo*, which share the same cytogenetic abnormalities, e.g., AML *de novo* with abnormalities of chromosome 5 or 7. In this chapter, we review the genetic characteristics of t-MN with an emphasis on defining the genetic pathways leading to t-MN with a del(5q).

## Cytogenetic Analyses:

**Figure 1.**
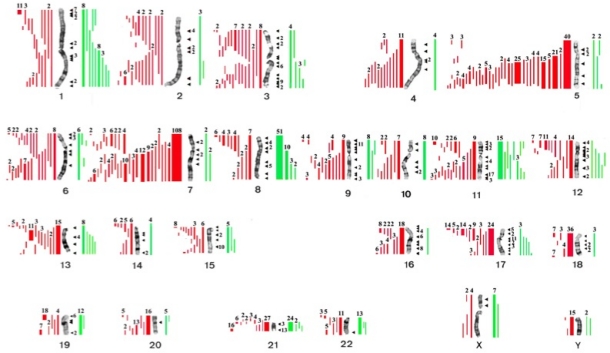
Cytogenetic abnormalities in patients with t-MN (N=386) in the University of Chicago series. Gain of chromosomal material is depicted by green bars to the right of each chromosome, and loss of chromosomal material is depicted by red bars. Numbers above the bars indicate the number of patients with this abnormality. Arrowheads identify the location of the breakpoints of structural rearrangements.

Table 1 summarizes the cytogenetic pattern in the recently updated University of Chicago series of 386 consecutive patients with t-MN. Of these, 349 (90.4%) had a clonal chromosomal abnormality, including 259 (67%) with a clonal abnormality leading to loss, deletion, or rearrangements of chromosomes 5 and/or 7 (referred to as del(5q)/t(5q) and -7/del(7q) herein),^4^ (Le Beau and Larson, unpublished data). Overall, 164 patients (42%) had abnormalities of chromosome 5, and 180 (47%) had abnormalities of chromosome 7. Of these patients, eighty-five patients had abnormalities of both chromosomes 5 and 7. A del(5q) was the most common structural abnormality. The pattern of numerical and structural abnormalities is shown in Figure 1, and illustrates that t-MN is associated with a complex karyotype, with a predominance of the loss of genetic material.

In other studies, we evaluated the cytogenetic pattern of 3,444 patients with primary MDS, AML *de novo*, or t-MN evaluated over the past 35 years by our Cancer Cytogenetics Laboratory. Of these, 553 (16%) patients had del(5q)/t(5q), and 597 (17.3%) had -7/del(7q) (Le Beau *et al*., unpublished data). Complex karyotypes were associated with abnormalities of chromosome 5, rather than chromosome 7. Recurring abnormalities observed at a high frequency (>20%) in patients with del(5q) included +8, and loss of 13q, 16q, 17p (40% of cases), chromosome 18, and 20q, which frequently occured in the same clone (Figure 1, and data not shown).

## Identification of Haploinsufficient Myeloid Suppressor Genes on 5q:

**Figure 2.**
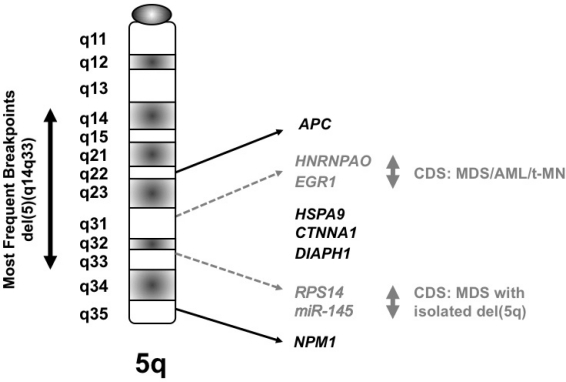
Idiogram of the long arm of chromosome 5 showing candidate genes within the CDSs as reported by various investigators. The proximal CDS in 5q31.2 was identified in MDS, AML and t-MN, whereas the distal CDS in 5q33.1 was identified in MDS with an isolated del(5q) (the 5q- Syndrome).

Several groups of investigators have defined a commonly deleted segment (CDS) on the long arm of chromosome 5 predicted to contain a myeloid tumor suppressor gene.^6^–^8^ Using cytogenetic and molecular analysis of MDS, AML and t-MN with a del(5q), we previously defined a region of 970 kilobase within 5q31.2, flanked by D5S479 and D5S500, that is deleted in all patients (Figure 2), and determined the genomic sequence of this region.^6^,^9^ In subsequent studies, we generated a transcript map of the CDS, and identified and cloned 19 genes;^9^ the CDS also contains one miRNA. The functions of the proteins encoded by these genes are diverse, and include the regulation of mitosis and the G2 checkpoint, transcriptional control, and translational regulation.

MDS with an isolated del(5q) (the 5q- Syndrome) is a distinct subtype of MDS, characterized by a macrocytic anemia, female predominance, and a favorable outcome, with a low risk of transformation to AML.^10^ Boultwood and colleagues identified a 1.5 Mb CDS within 5q33.1 between D5S413 and *GLRA1* .^8^ This region is distal to the CDS in 5q31.2 found in patients with AML with a del(5q). In summary, the existing data suggest that there are two non-overlapping CDSs: 5q31.2 in the more aggressive form of MDS, AML *de novo*, and t-MN, and 5q33.1 in the 5q- Syndrome (Figure 2).

Despite intense efforts, the identification of tumor suppressor genes (TSGs) on chromosomes 5 and 7 has been challenging. Molecular analysis of the 19 candidate genes within the CDS of 5q31.2 performed in our laboratory, and by Graubert *et al*., did not reveal inactivating mutations in the remaining alleles, nor was there evidence of transcriptional silencing (Godley and Le Beau, unpublished data).^9^,^11^ Similarly, molecular analysis of all 44 genes mapping to the CDS in 5q33.1 in the 5q- Syndrome did not reveal inactivating mutations.^8^ These observations are compatible with a haploinsufficiency model in which loss of one allele of the relevant gene(s) on 5q perturbs cell fate .^12^ A number of genes located on 5q, including RPS14 ,^13^ EGR1,^14^ NPM1,^15^ APC,^16^ CTNNA1,^17^ HSPA9,^18^ and DIAPH1,^19^ have been implicated in the development of myeloid disorders due to a gene dosage effect, and are reviewed briefly below. Together, these studies support a haploinsufficiency model, in which loss of a single allele of more than one gene on 5q contributes to the pathogenesis of t-MN with del(5q).

### RPS14:

The gene encoding RPS14, which is required for the processing of 18S pre-rRNA, is located at 5q33.1, and was identified as a candidate disease gene in the 5q- Syndrome.^13^ Downregulation of *RPS14* in CD34+ bone marrow cells blocks the differentiation of erythroid cells, and increases apoptosis in differentiating erythroid cells *in vitro*. Studies in a mouse model suggest that a TP53-dependent mechanism underlies this syndrome.^20^ Of interest, the ribosomal processing defect caused by haploinsufficiency of RPS14 in the 5q- Syndrome is highly analogous to the functional ribosomal defect seen in Diamond-Blackfan anemia. Other studies have shown that haploinsufficiency of two micro-RNAs (miRNAs) that are abundant in HSPCs, miR-145 and miR-146a, are encoded by sequences near the *RPS14* gene, and cooperate with loss of RPS14.^21^,^22^ The Toll-interleukin-1 receptor domain-containing adaptor protein (TIRAP) and tumor necrosis factor receptor-associated factor-6 (TRAF6) are respective targets of these miRNAs, implicating inappropriate activation of innate immune signals in the pathogenesis of the 5q-Syndrome.^21^ miR-145 also targets *FLI1*, a gene that promotes thrombocytopoiesis.^22^ Haploinsufficiency of miR-145 may account for several features of the 5q-Syndrome, including megakaryocytic dysplasia; however, neither *RPS14* nor miR-145 haploinsufficiency is predicted to confer clonal dominance.

### NPM1:

NPM1 is involved in ribosome biogenesis and centrosome duplication, and modulates the activity of the TP53 and CDKN2A tumor suppressors. *Npm1* heterozygous mice develop erythroid dysplasia with elevated mean corpuscular volume and red cell distribution width, normal red blood cell counts and hemoglobin (Hb) levels, and dysplastic megakaryocytes.^15^ However, the role of *NPM1* in the pathogenesis of MDS/AML is unclear, since *NPM1* is not deleted in most patients with a del(5q), nor have *NPM1* mutations been identified in patients with a del(5q).^23^

### CTNNA1:

Liu *et al.* showed that the α-catenin gene (*CTNNA1*) is down-regulated in stem and progenitor cells from MDS and AML patients with a del(5q) as compared to patients lacking del(5q), or normal HSPCs.^17^ *CTNNA1* is suppressed due to epigenetic silencing in HL-60 cells, a myeloid leukemia cell line used as a model for del(5q) leukemia. Reinduction of *CTNNA1* expression led to reduced proliferation, and an increased frequency of apoptosis, suggesting that down-regulation of α-catenin in HSPCs may contribute to transformation of myeloid cells in AML patients with a del(5q).^17^ However, analysis of mice with a conditional knockout of *Ctnna1* in hematopoietic cells (*Ctnna1+/−*), revealed no defects in hematopoiesis, or predisposition to myeloid neoplasms following mutagenesis with N-ethyl-nitrosourea (ENU).^24^

### HSPA9:

Heat shock protein A9 (HSPA9, also known as mortalin) is a highly conserved HSP70 family member that serves as a protein chaperone. Stimulation of hematopoietic progenitor cells with erythropoietin (EPO) results in upregulation of *HSPA9*, which may serve as a mediator of EPO signaling. The *HSPA9* gene is distal to the CDS in 5q31.2; however, one allele of this gene is deleted in the vast majority of cases. Using lentiviral-mediated knockdown in primary human hematopoietic cells and in a murine bone marrow-transplantation model, Chen *et al*. found that knockdown of *HSPA9* in human cells significantly delayed the maturation of erythroid precursors, increased apoptosis and decreased cell cycling; myeloid and megakaryocytic precursors were not affected.^18^ In the murine *Hspa9*-knockdown model, HSPCs, megakaryocyte/erythrocyte progenitors, erythroid precursors, and B lymphocytes were significantly reduced in number, suggesting that Hspa9 haploinsufficiency contributes to abnormal hematopoiesis. Nonetheless, additional cooperating gene mutations would be necessary for the pathogenesis of myeloid disorders and clonal dominance.

### DIAPH1:

The gene encoding Diaphanous-related formin, mDia1 (*DIAPH1*), maps to 5q31.3, between the CDSs identified in 5q31.2 and q33.1. mDia has critical roles in actin remodeling in cell division and in response to adhesive and migratory stimuli. *Diaph1+/−* and *Diaph1−/−* mice have normal hematopoiesis, but develop age-dependent myeloproliferative defects in a small percentage of mice.^19^ Eisenmann *et al*., have proposed that mDia1 acts as a node in a tumor-suppressor network that involves multiple 5q gene products (RPS14, EGR1, CTNNA1, and possibly APC). The network has the potential to sense dynamic changes in actin assembly, providing a homeostatic mechanism that serves to balance the regulation of growth control and differentiation in HSPCs. Although intriguing, this model awaits further experimental validation.

### APC:

APC (adenomatous polyposis coli) is a multifunctional tumor suppressor that is involved in the initiation and progression of colorectal cancer via regulation of the WNT signaling cascade. The *APC* gene is located at chromosome band 5q22.2, and is deleted in >95% of patients with a del(5q),^6^ raising the question of whether haploinsufficiency of *APC* contributes to the development of myeloid neoplasms with loss of 5q. Qian *et al*. employed the *Cre-loxP* system to inactivate *Apc* in hematopoietic cells *in vivo*.^25^ Conditional inactivation of *Apc in vivo* dramatically increased apoptosis and enhanced cell cycle entry of HSPCs, leading to their rapid disappearance and bone marrow failure. Conditional inactivation of a single allele of *Apc* in mice led to the development of severe anemia with macrocytosis and monocytosis.^16^ Further characterization of the erythroid lineage revealed that erythropoiesis was blocked at the early stages of differentiation. The short-term and long-term hematopoietic stem cell populations were expanded in *Apc-*heterozygous mice as compared to the control littermates; however, the HSPCs had a reduced capacity to regenerate hematopoiesis *in vivo* in the absence of a single allele of *Apc. Apc* heterozygous myeloid progenitor cells displayed an increased frequency of apoptosis, and decreased *in vitro* colony-forming capacity, recapitulating several characteristic features of myeloid neoplasms with a del(5q). These results indicated that haploinsufficiency of *Apc* impairs hematopoiesis, and raised the possibility that loss of function of *APC* contributes to the development of MDS and AML with a del(5q).

### EGR1:

The early growth response 1 gene (*EGR1)* encodes a member of the WT-1 family of transcription factors and contains 3 Cys_2_His_2_ Zn fingers that bind the GC-rich consensus sequences, GCG(G/T)GGGCG.^26^ In the mouse, *Egr1* has been shown to be an early response gene, and mediates the cellular response to growth factors, mitogens, and stress stimuli.^26^ *Egr1+/−*or *Egr1−/−* mouse embryonic fibroblasts bypass senescence and have immortalized growth characteristics, suggesting a role for Egr1 as a “gatekeeper” of p53-dependent growth regulation. *EGR1* has also been shown to act as a TSG in several human tumors, including breast carcinomas and non-small cell lung cancer.^27^ Recently, Egr1 has been shown to be a direct transcriptional regulator of many known TSGs, e.g., *Tp53, Cdkn1a/p21, Tgfb* and *Pten*.^27^

We characterized the hematopoietic potential of *WT*, *Egr1+/−,* and *Egr1−/−* mice, and found that heterozygous or homozygous loss of *Egr1* alone under normal physiological conditions does not affect the hematopoietic potential of murine marrow.^14^ However, Wagers and colleagues have shown that *Egr1*-deficient mice show spontaneous mobilization of HSPCs into the periphery, identifying Egr1 as a transcriptional regulator of stem cell migration.^28^ To investigate whether loss of *Egr1* cooperates with secondary mutations to induce leukemia in the mouse, we treated *WT*, *Egr1+/−,* and *Egr1−/−* mice with a single dose of 100mg/kg ENU at 4 weeks or 20 weeks of age. ENU was chosen because it is an alkylating agent, and may recapitulate the effects of alkylating agent chemotherapy in patients who develop t-MN. ENU-treated *Egr1+/−* and *Egr1−/−* mice developed a myeloproliferative disorder with ineffective erythropoiesis (MPD) at a high incidence. Our data suggests that loss of a single allele of *Egr1* cooperates with mutations induced by an alkylating agent in the development of malignant myeloid diseases in mice. Nevertheless, *Egr1* haploinsufficiency alone *in vivo* does not result in expansion of the HSPCs or abnormalities in adult hematopoiesis.^14^

**Figure 3.**
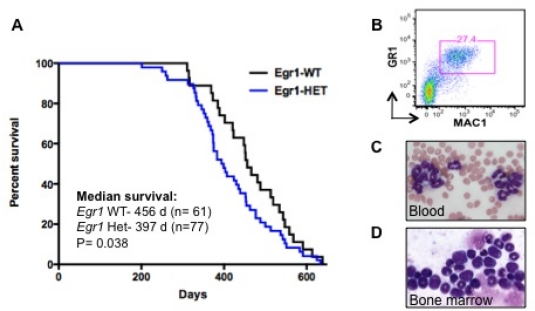
Retroviral insertional mutagenesis in *Egr1+/−* mice. *Egr1* WT (n=61) and *Egr1+/−* (n=77) neonatal mice were injected with MOL4070LTR retrovirus. (A) Survival curve for mice that developed myeloid neoplasms. (B) Flow cytometric analysis of spleen cells from a typical *Egr1+/−* diseased mouse revealed a Gr-1+Mac1+ myeloid neoplasm. (C, D) Wright-Giemsa-stained peripheral blood and bone marrow smears from a mouse with a myeloid neoplasm.

To identify genes that cooperate with loss of *Egr1* in murine leukemogenesis, we are using a forward genetic screen by retroviral insertional mutagenesis.^29^ We have injected cohorts of wild type (WT) (n=61) and *Egr1+/− (n=77)* neonatal mice with the MOL4070LTR retrovirus. WT and *Egr1*+/− mice developed disease at an onset of 7 mos. of age. *Egr1+/−*mice develop AML or an MPD-like leukemia with a shorter latency and at a higher overall frequency than WT littermate controls (median survival: *Egr1* WT 456 days, *Egr1* +/− 397 days, p=0.038) (Figure 3). Of note is that the incidence of myeloid disease is higher in *Egr1+/−* mice (68%) than in WT mice (49%), indicating that loss of one allele of *Egr1* shifts the disease spectrum from the more common lymphoid neoplasm to myeloid neoplasms. To identify cooperating cancer genes, we have cloned over 1500 retroviral integrations from myeloid neoplasms developing in 11 *Egr1* WT and 19 *Egr1+/−* mice. Ligation-mediated polymerase chain reaction was used to identify retroviral integrations at 30 common integration sites. The most common integration site was upstream of the *Evi1* gene. Of particular interest, 16/33 (48%) of *Egr1*+/− mice, as compared to 5/17 (29%) of *Egr1* WT mice had elevated *Evi1* expression in spleen cells from diseased mice, which correlated with retroviral integrations upstream of *Evi1*.

*EVI1* is overexpressed in AMLs, and is associated with a poor prognosis. Activation of *EVI1* in hematopoietic cells can occur by juxtaposition of the gene to enhancer elements of the ribophorin gene located at 3q21 as a result of the inv(3)(q21q26.2)/t(3;3)(q21;q26.2) and, in the t(3;21)(q26.2;q22), as part of the fusion *RUNX1/EVI1* mRNA transcribed from the der(3) chromosome. *EVI* is also overexpressed in 70% of AMLs with -7/del(7q), as well as in AMLs with translocations involving *MLL* at 11q23, and in many cases with a complex karyotype.^30^,^31^

EVI1 encodes a transcription factor that contains a seven-zinc-finger domain at the N-terminal end, a three-finger domain in the central part of the molecule, and an acidic domain distal to the second group of zinc fingers. EVI1 interacts with a number of transcriptional and epigenetic regulators (CREBBP, CTBP, HDAC, KAT2B (P/CAF), SMAD3, GATA1, GATA2, DNMT3A, and DNMT3B), and mediates chromatin modifications and DNA hypermethylation. Depending on it’s binding partners, EVI1 can act as a transcriptional activator to promote the proliferation of HSPCs, e.g., when bound to GATA2, or as a transcriptional repressor inhibiting erythroid differentiation, e.g., when bound to GATA1. In addition, EVI1 impairs myelopoiesis by de-regulation of multiple transcription factors, including RUNX1 and PU.1.^32^,^33^ This data raises the possibility that there are unique EVI1 interactions in each cytogenetic risk group that contribute to the development of disease. Engineered over-expression of *EVI1* in hematopoietic cells of transgenic mice or via bone marrow transplantation does not result in leukemia, indicating that additional cooperating genetic mutations are required for the pathogenesis of myeloid neoplasms. In the case of del(5q), haploinsufficiency of EGR1 in concert with high EVI1 may act to promote proliferation and self-renewal of HSPCs as well as to disrupt erythroid and myeloid differentiation.

### HNRNPA0:

A core set of genes has been identified as “master regulators” of myeloid differentiation. At the level of the granulocyte-monocyte progenitor, overall fate determination involves a balance of two opposing forces: PU.1 promotes monocytic differentiation, whereas CEBPA promotes granulocytic differentiation. Monocytic differentiation is promoted by the PU.1-induced transcription factors EGR2 and NAB2, whereas granulocytic differentiation is promoted by GFI1. EGR1/2 and NAB2 have been found to suppress the expression of *GFI1* and its downstream targets; conversely, GFI1 suppresses *EGR1, EGR2* and *NAB2*.^34^ In the context of the del(5q), EGR1 haploinsufficiency would be expected to deregulate myeloid cell differentiation, favoring granulocytic over monocytic differentiation. The *HNRNPA0* gene is also located within the CDS of 5q31.2, and is expressed at reduced levels in CD34+ cells from patients with MDS characterized by a del(5q) (Young and Le Beau, unpublished data). The HNRNPA0 protein is a member of the hnRNPA/B family of RNA-binding proteins, and has been shown to regulate transcript stability via binding to the AU-rich element of mRNAs. Using shRNAs in mouse hematopoietic cells, we demonstrated that knockdown of *Hnrnpa0* leads to a decrease in the stability of *Egr2* transcripts (Young and Le Beau, unpublished data). Thus, loss of a single allele of *EGR1* and *HNRNPA0* as a result of a del(5q) may lead to a synergistic disruption of EGR1/2 activity during leukemogenesis.

## Alterations in Gene Function:

A growing body of evidence suggests that mutations of multiple genes are involved in the pathogenesis and progression of t-MN. The involved genes fall into two main classes, namely, genes encoding hematopoietic transcription factors or proteins that regulate cytokine signaling pathways (Table 2). The RAS signaling cascade is downstream of a number of activated cytokine receptors, including the FLT3, IL3, and GM-CSF receptors; thus, this signaling pathway plays a pivotal role in hematopoiesis. Constitutively activating point mutations of *NRAS,* typically involving codons 12, 13, or 61, have been detected at high frequency in hematological malignancies (10–15% in t-MN).^35^ Mutations of the FMS-like tyrosine kinase 3 (*FLT3*) gene, including both point mutations within the tyrosine kinase domain and internal tandem duplications (ITDs), are among the most common genetic changes seen in AML *de novo* (15–35% of cases), but are less common in t-MN (0–12%).^35^,^36^ Mutations of *NPM1* also occur frequently in AML (35% of adult cases), but are less frequent in patients with recurring cytogenetic abnormalities, and in t-MN (4–10%). Of note, the *NPM1* gene located at 5q35 is not mutated in MDS with a del(5q).^15^

The Runt-related transcription factor 1 gene (*RUNX1*), also known as *AML1*, encodes the DNA-binding subunit of the heterodimeric core-binding factor (CBF) complex, which is essential for definitive hematopoiesis. Point mutations in the *RUNX1* Runt (DNA-binding) domain have been reported in AML and MDS (10–15%), particularly in MDS secondary to atomic bomb radiation exposure or treatment. Similarly, the incidence is higher in t-MN (15–30%).^37^ Moreover, *RUNX1* mutations are associated with activating mutations of the RAS pathway, -7/del(7q), and a shorter overall survival.

The *TP53* tumor suppressor gene encodes an essential checkpoint protein that monitors the integrity of the genome, and arrests cell cycle progression in response to DNA damage. Mutations of *TP53* are observed in primary MDS and AML *de novo* (5–10%) and, more commonly, in t-MN (25–30%).^35^,^38^ The spectrum of mutations includes missense mutations in exons 4–8, as well as loss of the wild type allele, typically as a result of a cytogenetic abnormality of 17p. In t-MN, *TP53* mutations are associated with -5/del(5q) and a complex karyotype.

The role of epigenetic changes in the pathogenesis and treatment of MDS and AML is becoming increasingly important. Transcriptional silencing via DNA methylation of the *CDKN2B* (*p15^INK4B^*) gene is observed in a high percentage of patients with t-MN, and is associated with -7/del(7q), and a poor prognosis.^39^,^40^ Other genes that may be affected by DNA methylation include the *CTNNA1* gene on 5q.

## Transcriptome Sequencing of t-MN:

To identify expressed genetic variants that distinguish the two subtypes of t-MN, we have used next generation sequencing of the mRNA transcriptome of primary patient leukemia samples. This technology has become significantly faster and cheaper,^41^ and has several advantages over other systems-level genomic approaches. Although whole genome sequencing of two *de novo* AML leukemias identified several mutations,^42^,^43^ mRNA sequencing enables analysis across a larger and more diverse set of samples at a fraction of the cost and time. Transcriptome sequencing better distinguishes expression levels and mRNA isoforms as compared to microarray analysis,^44^,^45^ and enables identification of cryptic gene fusions that cannot be detected by cytogenetic analysis.^46^ In addition to expression data, this method enables genotyping of the expressed coding region of the genome to identify inherited polymorphisms and somatic mutations.^47^

We hypothesized that the two t-MN subgroups, alkylating agent-related and topoisomerase II inhibitor-related, have unique, genome-wide patterns of transcriptionally expressed genetic variation that drive leukemogenesis. To test this, we employed Illumina paired-end technology to sequence the mRNA from the leukemia cells of 23 patients with t-MN (McNerney *et al*., unpublished data). These are comprised of 13 samples with del(5q) or -7/del(7q), or with complex karyotypes, and 12 cases with other karyotypes, including a normal karyotype or a balanced translocation. In addition, leukemia cells from 13 patients with AML *de novo* with a spectrum of cytogenetic abnormalities comparable to the t-MN samples were similarly analyzed.

Interestingly, there are significant differences between t-MN and AML *de novo* samples at the level of gene expression. t-MN samples exhibit different gene expression patterns as well as variable usage of transcript isoforms. This implies that there are underlying genetic differences between t-MN and AML *de novo*, consistent with the fundamental biological differences between these two diseases. In addition, there is significant differential gene expression between leukemias with abnormalities of chromosomes 5 and/or 7, as compared to those with other cytogenetic patterns. By comparing RNA-sequencing data to genome wide copy number variation using single nucleotide polymorphism arrays, we are identifying those genes (genome-wide as well as those mapping to chromosomes 5 and 7) that exhibit expression level differences secondary to DNA-dosage effects.

Using the RNA-sequencing data to genotype the samples, over 13,000 single nucleotide variants (SNVs) have been identified per sample, which is similar in number to other cancer samples. Phenotypically relevant SNVs are prioritized by population-level allele frequency estimates, evolutionary constraint estimates, and the predicted coding region change. This analysis yields approximately 200 rare, predicted deleterious SNVs per sample. Some of these variants include mutations that were previously reported in AML, such as mutations involving the *FLT3*, *TP53*, *RUNX1*, and *TET2* genes. However, mutations have not been reported previously in t-MN in most of the genes containing these variants, and many frequently occur in the same gene or pathway in multiple samples. A subset of the genes carry rare, deleterious variants, which occur preferentially in leukemias with abnormalities of chromosome 5 and/or 7. Intriguingly, the vast majority of rare, deleterious variants are inherited, which may guide us towards defining genetic predispositions to malignancy, in general, and to t-MN in particular. It is expected that genome-wide analyses across a large number of samples will result in the identification of the spectrum and frequency of expressed genetic variants in t-MN subtypes and the clinical impact of deleterious variants.

## Models for the Pathogenesis of t-MN:

**Figure 4.**
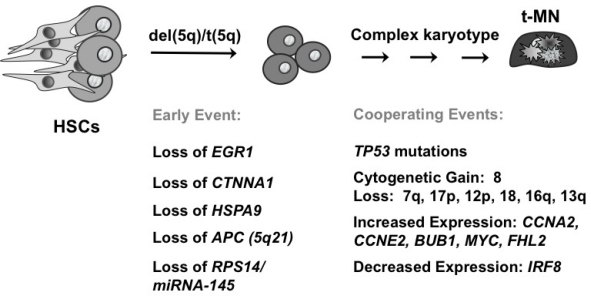
Cooperating genetic mutations leading to t-MN with a del(5q).

Extensive experimental evidence indicates that more than one mutation is required for the pathogenesis of hematological malignant diseases. Moreover, these mutations cooperate to confer a proliferative and/or antiapoptotic activity, as well as impair normal differentiation pathways. Haploinsufficiency for a gene(s) on 7q and 5q is likely to be an initiating mutation. Pedersen-Bjergaard and colleagues have proposed 8 different pathways that are involved in progression to t-MN.^48^ Pathway I consists of patients who have abnormalities of chromosome 7, without chromosome 5 abnormalities. These patients often present with mutations of the RAS pathway (*KRAS, NRAS, NF1, PTPN11*), and methylation silencing of *p15 (CDKN2B),* and they have a poor prognosis. Loss of *TAL1*, *GATA1*, and *EKLF* expression in t-MN with a -7/del(7q) may result in impaired differentiation, whereas overexpression of *FLT3*, *PIK3C2B*, and *BCL2* result in a proliferative/survival advantage.^49^ Pathway II comprises patients with a del(5q) with or without abnormalities of chromosome 7, and a poor prognosis (Figure 4). Haploinsufficiency of multiple, cooperating genes on 5q is likely to be the initiating event. Genomic instability and complex karyotypes with gain of chromosome 8, and loss of 12p, 13q, 16q, 17p (*TP53* locus), chromosome 18, and 20q, as well as mutations of *TP53* are often observed in this subgroup. In t-MN patients with del(5q), loss of expression of *IRF8* may lead to impaired differentiation and/or a survival advantage, whereas increased expression of cell cycle regulatory proteins (CCNA2, CCNE2, CDC2) would result in a proliferative advantage.^49^

Pathway III consists of patients with translocations of 11q23. Alterations of pathway IV convey the best prognosis for patients with t-MN, and include the t(8;21) or inv(16). Pathway V comprises patients who present with therapy-related acute promyelocytic leukemia with the t(15;17) resulting in the *PML-RARA* fusion and a good prognosis. Pathway VI involves balanced translocations of *NUP98* at 11p15. Pathway VII includes t-MN with a normal karyotype. Recently, internal tandem duplications of *FLT3* and *MLL,* and *NPM1* mutations have been described in a few of these patients. Pathway VIII includes patients with other chromosomal abnormalities. New technologies, such as high-throughput genomics technologies, will facilitate further delineation of the genetic pathways leading to t-MN.

## Concluding Remarks:

t-MN remains one of the most adverse complications of successful therapy for a variety of malignant and non-malignant conditions. The factors that place individual patients at risk are beginning to be elucidated, and are critical for risk-assessment, to allow individualized therapy directed at minimizing the development of this disease. Moreover, characterizing the genetic pathways that give rise to t-MN will lead to a greater understanding of genetic susceptibility to this disease, as well as the molecular features of the disease and, ultimately, may lead to more targeted therapies for its treatment.

## Figures and Tables

**Table 1.** Cytogenetic abnormalities in 386 patients with t-MN.

**Karyotype**	**No. of patients (%)**
Normal karyotype	37 (9.6)
Clonal abnormalities	349 (90.4)
Clonal abnormalities of chromosomes 5, 7, or both (+/− other abnormalities)	259 (67)
Abnormal chromosome 5 only	79 (20)
Abnormal chromosome 7 only	95 (25)
Abnormal chromosomes 5 and 7	85 (22)
Recurring balanced rearrangements ^a^	41 (10.6)
t(11q23)	16 (3)
t(3;21) or t(8;21) or t(21q22)	10 (3)
t(15;17)	8 (2)
inv(16)	7 (2)
Recurring unbalanced abnormalities ^a^	21 (5)
+8	10 (3)
-13/del(13q)	3 (1)
-Y,+11,del(11q),del(20q),+21	8 (2)
Other clonal abnormalities	29 (7.5)

a: One patient with an abnormality of chromosome 5 and t(3;21), and is listed twice in the table.

**Table 2.** Frequency of gene mutations in AML *de novo* and t-MN.

**Mutated Gene**	**AML *de novo***	**t-MN**
***FLT3 (ITD)***	35%	0–10%
***FLT3 (TKD)***	9%	<1%
***NRAS***	10–15%	10%
***KIT****^D816^*	∼5%	NA
***MLL (ITD)***	3%	2–3%
***RUNX1***	10–15%	15–30%
***TP53***	10%	25–30%
***PTPN11***	∼2%	3%
***NPM1***	35–50%	4–10%*
***CEBPA***	6–15%	Rare
***JAK2****^V617F^*	2–5%	2–5%
***IDH1/2***	15%	<10%
***TET2***	5–15%	15–30%
***ASXL***	5%	15–40%
***EZH2***	5%	6–25%

*NPM1* mutations are associated with a normal karyotype in t-MN.
